# Severe Phenotype in a Patient With Homozygous 15q21.2 Microdeletion Involving *BCL2L10*, *GNB5*, and *MYO5C* Genes, Resembling Infantile Developmental Disorder With Cardiac Arrhythmias (IDDCA)

**DOI:** 10.3389/fgene.2020.00399

**Published:** 2020-05-13

**Authors:** Francesca L. Sciacca, Claudia Ciaccio, Federica Fontana, Camilla Strano, Francesca Gilardoni, Chiara Pantaleoni, Stefano D’Arrigo

**Affiliations:** ^1^Neurological Biochemistry and Neuropharmacology Unit, Laboratory of Cytogenetic, Department of Diagnostic and Technology, Fondazione IRCCS Istituto Neurologico Carlo Besta, Milan, Italy; ^2^Developmental Neurology Unit, Fondazione IRCCS Istituto Neurologico Carlo Besta, Milan, Italy

**Keywords:** 15q21.2 microdeletion, infantile developmental disorder with cardiac arrhythmias (IDDCA), GNB5, BCL2L10, MYO5C, epileptic encephalopathy, neurodevelopmental diseases

## Abstract

Homozygous and compound heterozygous mutations in *GNB5* gene have been associated with a wide spectrum of clinical presentations, ranging from neurodevelopmental issues with or without cardiac arrhythmia (LADCI) to severe developmental delay with epileptic encephalopathy, retinal dystrophy, and heart rhythm abnormalities (IDDCA). While missense or missense/non-sense mutations usually lead to milder form, the biallelic loss of function of *GNB*5 gene causes the severe multisystemic IDDCA phenotype. So far, only 27 patients have been described with *GNB5*-associated disease. We report the first case of a patient carrying a homozygous 15q21.2 microdeletion, encompassing *GNB5* and the two contiguous genes *BCL2L10* and *MYO5C*. The clinical features of the child are consistent with the severe IDDCA phenotype, thus confirming the *GNB5* loss-of-function mechanism in determining such presentation of the disease.

## Introduction

Chromosomal aberrations are a common cause of developmental delay/intellectual disability (DD/ID) and congenital malformations (Stankievic and Beaudet, 2007; Sagoo et al., 2009). We now know that as many as 30–50% of cases of ID with or without other pathological features are caused by genetic or chromosomal anomalies (Cooper et al., 2011; Schaaf et al., 2011; Srivastava et al., 2019). In the present report, we describe a male patient affected by profound development delay with absence of motor and language acquisition, early onset epilepsy, bradycardia, nystagmus, visual impairment, and severe gastroesophageal reflux. Array-CGH analysis demonstrated the presence of a homozygous deletion in 15q21.2, spanning about 193 kb and involving *BCL2L*, *GNB5*, and *MYO5C* genes. Among these three genes, *GNB5* has been already associated with neurodevelopmental impairment and variable multisystemic dysfunction in 27 cases (Table 1).

**TABLE 1:** Summary of cases with *GNB5* mutations so far reported compared to the present case with homozygous microdeletion.

Lodder et al. (2016)	Family A II.1^§^	• c.249G > A (p.Asp84Valfs*52) pat	Severe DD, epilepsy, nystagmus, hypotonia, hyporeflexia, CA, GER – Brain MRI: normal
		• c.994C > T (p.Arg332*) mat
	Family A II.2^§^	• c.249G > A (p.Asp84Valfs*52) pat	Severe DD, epilepsy, nystagmus, retinopathy, hypotonia, hyporeflexia, CA, PFO, GER – Brain MRI: normal
		• c.994C > T (p.Arg332*) mat
	Family B II.1^§^	• c.249 + 1G > T (p.Asp84Leufs*31) pat	Severe DD, epilepsy, nystagmus, hypotonia evolving into spasticity, CA – Brain MRI: cerebral atrophy
		• c.249 + 1G > T (p.Asp84Leufs*31) mat
	Family C II.2	• c.249 + 3A > G (p.Asp84Valfs*31) pat	Severe DD, nystagmus, hypotonia, CA, GER
		• c.249 + 3A > G (p.Asp84Valfs*31) mat
	Family C II.3	• c.249 + 3A > G (p.Asp84Valfs*31) pat	DD, nystagmus, hypotonia, CA, GER
		• c.249 + 3A > G (p.Asp84Valfs*31) mat
	Family D II.2^§^	• c.906C > G (p.Tyr302*) pat	Severe DD, epilepsy, nystagmus, hypotonia, CA, GER – Brain MRI: normal
		• c.906C > G (p.Tyr302*) mat
	Family E II.1	• c.242C > T (p.Ser81Leu) pat	Mild ID, language delay, CA
		• c.242C > T (p.Ser81Leu) mat
	Family E II.2	• c.242C > T (p.Ser81Leu) pat	Mild ID, language delay, CA
		• c.242C > T (p.Ser81Leu) mat
	Family F II.1	• c.242C > T (p.Ser81Leu) pat	Mild ID, CA
		• c.242C > T (p.Ser81Leu) mat
Shamseldin et al. (2016)	V:1	• c.242C > T (p.Ser81Leu) pat	Severe language delay, ADHD
		• c.242C > T (p.Ser81Leu) mat
	V:2	• c.242C > T (p.Ser81Leu) pat	Severe language delay, ADHD
		• c.242C > T (p.Ser81Leu) mat
	V:3	• c.242C > T (p.Ser81Leu) pat	Severe language delay, motor delay
		• c.242C > T (p.Ser81Leu) mat
	IV:1	• c.242C > T (p.Ser81Leu) pat	Severe language delay, motor delay, hypotonia
		• c.242C > T (p.Ser81Leu) mat
	IV:6	• c.242C > T (p.Ser81Leu) pat	Severe language delay, mild motor delay, ADHD
		• c.242C > T (p.Ser81Leu) mat
Turkdogan et al. (2017)	V.1	• c.355delG (p.Ala119Profs*16) pat	Severe DD, epilepsy, nystagmus, retinopathy, hypotonia, autism, CA, microbrachycephaly
		• c.355delG (p.Ala119Profs*16) mat
	IV.8	• c.355delG (p.Ala119Profs*16) pat	Severe DD, epilepsy, nystagmus, hypotonia, CA, microbrachycephaly – Deceased 5m
		• c.355delG (p.Ala119Profs*16) mat
	IV.11	• c.355delG (p.Ala119Profs*16) pat	Severe DD, epilepsy, nystagmus, hypotonia, CA, microbrachycephaly – Deceased 7m
		• c.355delG (p.Ala119Profs*16) mat
	IV.12	• c.355delG (p.Ala119Profs*16) pat	Severe DD, epilepsy, nystagmus, hypotonia, CA, microbrachycephaly – Deceased 8m
		• c.355delG (p.Ala119Profs*16) mat
	IV.13	• c.355delG (p.Ala119Profs*16) pat	Severe DD, epilepsy, nystagmus, hypotonia, CA, microbrachycephaly – Deceased 8m
		• c.355delG (p.Ala119Profs*16) mat
	IV.14	• c.355delG (p.Ala119Profs*16) pat	Severe DD, epilepsy, nystagmus, retinopathy, hypotonia, autism, CA, microbrachycephaly – Brain MRI: normal
		• c.355delG (p.Ala119Profs*16) mat
Vernon et al. (2018)		• c.737G > A (p.Arg246Gln) pat	Severe DD, nystagmus, retinopathy, central hypotonia/intermittent extremities hypertonia, upper limbs involuntary movements, CA, GER, left ear hearing loss, laryngomalacia – Brain MRI: thin corpus callosum
		• c.222_226delTAAGA (p.Asp74Glufs*52) mat
Malerba et al. (2018)		• c.222_226delTAAGA (p.Asp7Glufs*52) pat	Mild ID, language delay, strabismus, CA, hypotonia
		• c.242C > T (p.Ser81Leu) mat
Shao et al. (2019)^§^		• c.906C > A (p.Tyr302*) pat	Severe DD, epilepsy, retinopathy, hypotonia, hyporeflexia, CA, central sleep apnea – Brain MRI: long posterior corpus callosum
		• c.906C > A (p.Tyr302*) mat
Poke et al. (2019)	P1	• c.136delG (p.Glu46fs*8) pat	Severe DD, epilepsy, visual impairment, hypotonia, contractures, CA – Brain MRI: normal
		• c.136delG (p.Glu46fs*8) mat
	P3	• c.242C > A (p.Ser81*) pat	Severe DD, epilepsy, nystagmus, hypotonia – Deceased 13y – Brain MRI: normal
		• c.242C > A (p.Ser81*) mat
	P4	• c.242C > A (p.Ser81*) pat	Severe DD, epilepsy, nystagmus, retinopathy, hypotonia, CA – Brain MRI: mild ventricular asymmetry
		• c.242C > A (p.Ser81*) mat
	P8	• c.906C > G (p.Tyr302*) pat	Severe DD, epilepsy, nystagmus, retinopathy, hypotonia, hyporeflexia, CA – Brain MRI: normal
		• c.906C > G (p.Tyr302*) mat
Current case		Arr[Hg19]15q21.2:(52385564_52579282)x0 mat, pat	Severe DD, epilepsy, nystagmus, retinopathy, central hypotonia/intermittent extremity hypertonia, CA, GER, central sleep apnea – Brain MRI: cerebral and cerebellar cortical atrophy

*DD, Developmental Delay; ID, Intellectual Disability; CA, Cardiac Arrhythmia; PFO, Patent Foramen Ovale; GER, GastroEsophageal Reflux; ADHD, Attention Deficit/Hyperactivity Disorder; MRI, Magnetic Resonance Imaging; m, months/y, years. ^§^ Reported in Poke et al. (2019).*

*GNB5* encodes a β subunit of heterotrimeric GTP-binding proteins. Its transcript binds members of the R7 family of G-protein-signaling regulators (RGS), supporting their negative regulation of G protein-coupled receptor signaling. The R7 RGS family is widely expressed in the central nervous system, and *GNB5* transcript is involved in multiple signaling pathways in the brain. It has a central role in parasympathetic control of heart rate, neuronal development, motor function, and vision (Lodder et al., 2016). Recently, homozygous or compound heterozygous pathogenic variants in *GNB5* have been reported as the cause of an autosomal recessive multisystemic syndrome with a wide spectrum of clinical presentation, categorized under two distinct phenotypes. The final presentation depends on the severity of the G protein beta’s impaired function determined by the mutation. Specifically, homozygous carriers of missense variants, the most common being c.242 C > T p.(Ser81Leu), present a mild form characterized by language delay, cognitive impairment, attention-deficit/hyperactivity disorder (ADHD), with or without cardiac arrhythmia (LADCI, #617182) (Lodder et al., 2016; Shamseldin et al., 2016). Otherwise, homozygous carriers of non-functional alleles are affected by a severe form characterized by developmental delay evolving in severe intellectual disability with poor or absent speech, early epileptic encephalopathy, hypotonia, retinal abnormalities and sick sinus syndrome with bradycardia, escape beats and other arrhythmias in the absence of structural heart abnormalities (IDDCA, #617173) (Lodder et al., 2016; Turkdogan et al., 2017; Malerba et al., 2018; Vernon et al., 2018; Poke et al., 2019; Shao et al., 2019).

As this is the first case of homozygous deletion involving the *GNB5* gene previously associated to diseases with complex phenotype in patients with homozygous mutations, here, we describe our patient and discuss the implications for the diagnostic assessment.

## Case Report

Our patient is a male child, born from first cousin healthy parents of Egyptian ancestry; no remarkable issue was reported in family history, but the parental couple experienced five miscarriages before the patient was born. After an uneventful full-term pregnancy, he was born trough an emergency C-section, performed for fetopelvic disproportion and initial fetal distress. We were unable to access any documentation about neonatal parameters, but perinatal period was reported as physiological, except for mild jaundice and the evidence of minor heart defects (patent foramen ovale and ductus arteriosus, both later spontaneously closed). At the age of 6 months he experienced the onset of infantile spasm epilepsy, for which he was initially treated with valproic acid (VPA), discontinued at 3 years of age after remission of the symptoms; VPA was reintroduced 1 year later, when the child started manifesting major critical episodes characterized by hypertonia and upward gaze deviation, multiple times a day. No episodes have been reported since the introduction of a combination treatment with VPA and levetiracetam (LVT). His current phenotype is that of a severe neurodevelopmental impairment (he managed to acquire partial head control by 1 year of age but no further developmental milestones; language is completely absent), cortical blindness with subcontinuous erratic eye movements, and generalized epilepsy. Auxometric parameters are within the normal range (8 years old: height 120 cm, −0.3 SD; weight 19 kg, −1.6 SD; head circumference 50 cm, −1.5 SD), and he does not present any facial peculiar characteristics or somatic malformation. Electroencephalographic registration revealed severe disorganization with recurrent generalized epileptic anomalies, predominant on the frontal lobes. Brain MRI showed moderate bilateral enlargement of the ventricular system and cerebral sulci, suggestive of global supra and subtentorial atrophy. His heart rate was reduced at heart auscultation, and ECG registration confirmed the presence of a marked sinus bradycardia, with heart rate of 39; heart ultra sounds demonstrated the absence of structural anomalies, while at the Holter monitoring, significant sinus arrhythmia, mostly nocturnal, was found. A subsequent cardiac evaluation ruled out the need, at that moment, of further interventions for the bradycardia. Nocturnal polysomnography was also performed, showing periodic breathing with several desaturations and episodic bradypnea, suggestive of autonomic nervous system impairment. Furthermore, marked alterations, displayed by electroretinogram, indicated the presence of retinal dystrophy. The patient also suffers from severe gastroesophageal reflux disease.

### Array-CGH Analysis

DNA was extracted from peripheral blood using a specific kit (Gentra kit, Qiagen, Hilden, Germany). ACGH analyses were performed using the Cytosure Oligo ISCA180K platform, which comprises a research-validated collection of specific probes that enable reliable detection of CNVs with high resolution in regions associated to genetic disorders. Array design was performed by Oxford Gene Technology (OGT; Begbroke, Oxfordshire, United Kingdom) and manufactured by Agilent Technologies (Santa Clara, CA, United States). The DNA test was hybridized with sex-matched DNA from pooled controls (reference DNA; Promega, Madison, WI, United States), according to the manufacturer’s protocol. Hybridization was performed using MaiTaiTM Hybridization System (SciGene). After 20 h, CytoChip Oligo array was washed and scanned using InnoScan 710 Microarray Scanner (Innopsys, Carbonne, FR). Amplifications or deletions are revealed by green (Cy3) or red (Cy5) signals, due to unbalanced ratio between the two fluorophores. Data were analyzed using Cytosure interpret software (Oxford Gene Technology). Clinical interpretation of Array-CGH results are based on published literature and public databases (ENSEMBL, USBC, Database for Genetic Variants, DECIPHER, the Italian database of Troina) following Cytogenetic European and International Guidelines (Kearney et al., 2011; Hastings et al., 2012). Genomic coordinates are based on the February 2009 Human Genome Build (GRCh37/hg19).

## Results

**FIGURE 1:**
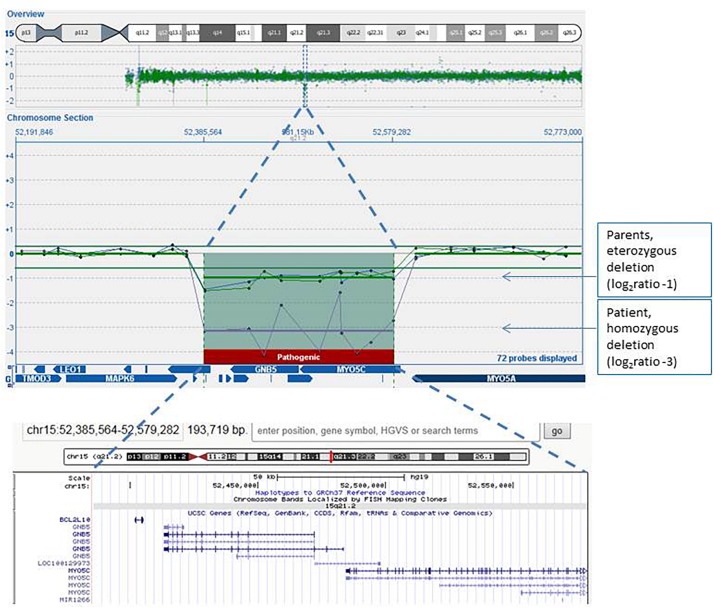
Array-CGH genomic profile (screenshot from the Cytosure software analyses) focused on the 15q21.2 chromosome region of the proband (purple line), his mother (green line), and his father (blue line). The log2 ratio of microdeletion of the patient was about −3, indicating homozygous deletion, while log2 ratios of the microdeletion carried by both parents were −1, indicating heterozygous loss of the region. Also indicated in the software figure is the gene content of the region: the same region with its gene content is also enlarged in the lower image, captured in the UCSC genome browser.

We screened copy number variations by Array-CGH, and we detected a homozygous deletion in 15q21.2. The proximal breakpoint is between nucleotide 52,366,562 and 52,385,564, and the distal breakpoint is between 52,579,282 and 52,602,756, thus spanning about 193–236 kb. The deleted region involved *BCL2L*, *GNB5*, and *MYO5C* genes (Figure 1). Both parents carried a heterozygous deletion overlapping with that and showed no clinical sign of disease.

No cases with similar homozygous microdeletion are present in public databases (Decipher, ClinVar Long Variants, ClinGen CNVs), while in the literature, 27 patients carrying *GNB5* homozygous or compound heterozygous mutations can be found (Lodder et al., 2016; Shamseldin et al., 2016; Turkdogan et al., 2017; Malerba et al., 2018; Vernon et al., 2018; Poke et al., 2019; Shao et al., 2019). MIM database indicates that mutations in *GNB5* are associated with autosomal recessive disorders: infantile developmental disorder with cardiac arrhythmias (IDDCA, MIM# 617173) and language delay and ADHD/cognitive impairment with or without cardiac arrhythmia (LADCI, MIM# 617182). IDDCA may occur with a severe phenotype, when caused by loss-of-function mutations in both alleles, or with less severe clinical features when caused by compound heterozygous non-sense/missense mutations (Shamseldin et al., 2016; Malerba et al., 2018; Vernon et al., 2018); LADCI is a mild/moderate form of disease, caused by missense mutations in both alleles.

The clinical features of our patient were almost completely overlapping with severe phenotype of IDDCA, so we can infer that homozygous *GNB5* deletions determine the syndrome, as well as homozygous loss-of-function mutations of the gene.

## Discussion

Infantile developmental disorder with cardiac arrhythmias (IDDCA) is an autosomal recessive multisystem disorder characterized by cognitive impairment, poor or absent speech, delayed motor development, seizures, hypotonia, retinal disease, nystagmus, sinus node dysfunction, and gastro-esophageal reflux. This disorder is severe and is caused by homozygous loss-of-function mutations in the *GNB5* gene that encodes for one out of five variants of the beta subunit of the G protein (G protein β5) (Lodder et al., 2016). The IDDCA phenotype is very similar to that of our patient, so that *GNB5* must be considered the main factor in determining the clinical features of the child, given its phenotypic overlap with patients carrying *GNB5* null mutations.

G protein β5 is involved in regulation of a plethora of cellular activities (Simonds and Zhang, 2000). Human *GNB5* is expressed in the brain, pancreas, kidney, heart (Jones et al., 1998) and retina (Watson et al., 1996). Within the brain, the highest expression is found in the cerebellum, cerebral cortex, occipital pole, frontal lobe, temporal lobe, and caudate putamen, while the lowest expression was in the corpus callosum and spinal cord (Jones et al., 1998). It is a part of the neurotransmitter signaling cascade of the G protein-coupled receptor and plays a crucial role in psychiatric functions (Catapano and Manji, 2007; Meye et al., 2014), heart rate regulation (Posokhova et al., 2010), motor functions (Zhang et al., 2011), and vision (Shao et al., 2019). The mouse *Gnb5* knockout model shows high mortality rate (Chen et al., 2003; Zhang et al., 2011), somatic runty at birth, and then a small body size, significant developmental milestone delays, abnormal gate, balance and motor learning, hyperactivity, delayed Purkinje cell development, reduced dendritic arborization, abnormal hippocampal development, and changes in transcription levels of several genes (Zhang et al., 2011). Both the knockout mice and the patients with homozygous loss-of-function mutations show phenotypic overlap with our patient: such considerations strongly suggest that *GNB5* deletion plays a predominant role in defining the clinical presentation of our child.

Despite the seriousness of the phenotype, neuroradiological study is normal in most patients reported in literature, or it sometimes shows minor dysmorphic anomalies (Lodder et al., 2016; Vernon et al., 2018; Poke et al., 2019). In our patient, a diffuse mild cerebral and cerebellar atrophy was found; such sign has been developed only by one patient, reported in both Lodder et al. (2016) and Poke et al. (2019) papers. We also underline the detection in our patient of nocturnal periodic breathing with numerous desaturations and episodic bradypnea. Sleep apneas have only been reported once by Shao et al. (2019): we can confirm the possible presence of such comorbidity in IDCCA that, along with bradycardia and arrhythmias, points out an autonomic nervous system impairment resulting from *GNB5* loss of function.

*BCL2L10* and *MYO5C* genes are also involved in the microdeletion of our case, so that we tried to understand if the homozygous deletion of these genes could modify the phenotype.

*BCL2L10* codes for a protein widely expressed in adult tissues, preferentially in the lungs, liver, and kidneys. Overexpression of *BCL2L10* suppresses cell apoptosis (Ke et al., 2001). No data are available on the effect of homozygous loss of this gene, so that we can only speculate that the lack of this pro-apoptotic gene could deregulate cell survival process and increase the risk of cancer incidence. Appropriate clinical follow up will be set up for our patient.

*MYO5C* codifies for type Vc myosin protein, which mediates the transport of several protein complexes such as organelles, vesicles, and mRNAs along actin cables (Hammer and Sellers, 2012). Tissue expression and object transport specificity are peculiar of each myosin V: Myosin Vc is mostly expressed in epithelial and glandular tissues (Rodriguez and Cheney, 2002; Jacobs et al., 2009). So far, no mutation in *MYO5C* has been linked to a heritable syndrome. The protein myosin Vc is implicated in transport of secretory vesicles. Together with other proteins of the Rab family, myosin Vc is involved in the maturation of melanosomes in skin melanocytes and in retinal pigmented epithelial cells (Bultema et al., 2014), as well as in transferrin trafficking (Rodriguez and Cheney, 2002). Basing on the current knowledge on the gene, no correlation between the biallelic loss of *MYO5C* protein product and the clinical phenotype of our patient can be hypothesized.

## Conclusion

This report describes the first ever reported case carrying a homozygous 15q21.2 deletion encompassing *GNB5* and showing a phenotype consistent with that of the patients with homozygous loss-of-function mutation of the gene. We can, therefore, confirm the correlation between the phenotype severity and the absence of G protein β5, as proposed by previous reports that associated homozygous null mutations with IDDCA severe phenotype.

## Data Availability Statement

The patient’s dataset is available in the public database DECIPHER (case number 411168).

## Ethics Statement

Written informed consent was obtained from the minor(s)’ legal guardian/next of kin for the publication of any potentially identifiable images or data included in this manuscript.

## Author Contributions

FS interpreted the Array-CGH analysis, conceived and co-wrote the study. CC and SD’A performed the clinical assessment and follow-up of the patient and co-wrote the manuscript. CS helped in studying the case and drafting the manuscript. FF and FG performed the laboratory tests. CP reviewed the manuscript for intellectual content and supervised the work.

## Conflict of Interest

The authors declare that the research was conducted in the absence of any commercial or financial relationships that could be construed as a potential conflict of interest.

## References

[B1] Bultema J. J. Boyle J. A. Malenke P. B. Martin F. E. Dell’Angelica E. C. Cheney R. E. (2014). Myosin Vc interacts with Rab32 and Rab38 proteins and works in the biogenesis and secretion of melanosomes. *J. Biol. Chem.* 289 33513–33528. 10.1074/jbc.M114.578948 25324551 PMC4246105

[B2] Catapano L. A. Manji H. K. (2007). G protein-coupled receptors in major psychiatric disorders. *Biochim. Biophys. Acta* 1768 976–993. 10.1016/j.bbamem.2006.09.025 17078926 PMC2366056

[B3] Chen C. K. Eversole-Cire P. Zhang H. Mancino V. Chen Y. J. He W. (2003). Instability of GGL domain-containing RGS proteins in mice lacking the G protein beta-subunit Gbeta5. *Proc. Natl. Acad. Sci. U.S.A.* 100 6604–6609. 10.1073/pnas.0631825100 12738888 PMC164494

[B4] Cooper G. M. Coe B. P. Santhosh Girirajan S. Rosenfeld J. A. Vu T. H. Carl Baker C. (2011). A copy number variation morbidity map of developmental delay. *Nat. Genet.* 43 838–846. 10.1038/ng.909 21841781 PMC3171215

[B5] Hammer J. A. III Sellers J. R. (2012). Walking to work: roles for class V myosins as cargo transporters. *Nat. Rev. Mol. Cell. Biol.* 13 13–26. 10.1038/nrm3248 22146746

[B6] Hastings R. Howell R. Dagna Bricarelli F. Kristoffersson U. Cavani S. (2012). Specific constitutional cytogenetic guidelines of european cytogeneticist association E.C.A. *Eur. Cytogenet. Assoc. Newsl.* 2012 9–20.

[B7] Jacobs D. T. Weigert R. Grode K. D. Donaldson J. G. Cheney R. E. (2009). Myosin Vc is a molecular motor that functions in secretory granule trafficking. *Mol. Biol. Cell.* 20 4471–4488. 10.1091/mbc.E08-08-0865 19741097 PMC2770936

[B8] Jones P. J. Lombardi S. J. Cockett M. I. (1998). Cloning and tissue distribution of the human G protein beta 5 cDNA. *Biochim. Biophys. Acta* 1402 288–291. 10.1016/s0167-4889(98)00017-2 9606987

[B9] Ke N. Godzik A. Reed J. C. (2001). Bcl-B, a novel Bcl-2 family member that differentially binds and regulates Bax and Bak. *J. Biol. Chem.* 27 12481–12484. 10.1074/jbc.C000871200 11278245

[B10] Kearney H. M. Thorland E. C. Brown K. K. Quintero-Rivera F. South S. T. Working Group of the American College of Medical Genetics Laboratory Quality Assurance Committee (2011). American College of Medical Genetics standard and Guidelines for interpretation and reporting of postnatal constitutional of copy number variants. *Genet. Med.* 13 680–685. 10.1097/GIM.0b013e3182217a3a 21681106

[B11] Lodder E. M. De Nittis P. Koopman C. D. Wiszniewski W. Fischinger Moura de Souza C. Najim Lahrouchi N. (2016). GNB5 mutations cause an autosomal-recessive multisystem syndrome with sinus bradycardia and cognitive disability. *Am. J. Hum. Genet.* 99 704–710. 10.1016/j.ajhg.2016.06.025 27523599 PMC5010642

[B12] Malerba N. Towner S. Keating K. Squeo G. M. Wilson W. Merla G. (2018). A NGS-targeted autism/ID panel reveals compound heterozygous GNB5 variants in a novel patient. *Front. Genet.* 6:626. 10.3389/fgene.2018.00626 30631341 PMC6315145

[B13] Meye F. J. Ramakers G. M. J. Adan R. A. H. (2014). The vital role of constitutive GPCR activity in the mesolimbic dopamine system. *Transl. Psychiatry* 4:e361. 10.1038/tp.2013.130 24518399 PMC3944632

[B14] Poke G. King C. Muir A. de Valles-Ibáñez G. Germano M. Moura de Souza C. F. (2019). The epileptology of GNB5 encephalopathy. *Epilepsia* 60 e121–e127. 10.1111/epi.16372 31631344

[B15] Posokhova E. Wydeven N. Allen K. L. Wickman K. Kirill A. Martemyanov K. A. (2010). RGS6/Gβ5 complex accelerates IKACh gating kinetics in atrial myocytes and modulates parasympathetic regulation of heart rate. *Circ. Res.* 107 1350–1354. 10.1161/CIRCRESAHA.110.224212 20884879 PMC3014848

[B16] Rodriguez O. C. Cheney R. E. (2002). Human myosin-Vc is a novel class V myosin expressed in epithelial cells. *J. Cell. Sci.* 115 991–1004. 11870218 10.1242/jcs.115.5.991

[B17] Sagoo G. S. Butterworth A. S. Sanderson S. Shaw-Smith C. Higgins J. P. T. Burton H. (2009). Array CGH in patients with learning disability (mental retardation) and congenital anomalies: updated systematic review and meta-analysis of 19 studies and 13,926 subjects. *Genet. Med.* 11 139–146. 10.1097/GIM.0b013e318194ee8f 19367186

[B18] Schaaf C. P. Wiszniewska J. Beaudet A. L. (2011). Copy number and SNP arrays in clinical diagnosis. *Annu. Rev. Genomics Hum. Genet.* 12 25–51. 10.1146/annurev-genom-092010-110715 21801020

[B19] Shamseldin H. E. Masuho I. Alenizi A. Alyamani S. Patil D. N. Ibrahim N. (2016). GNB5 mutation causes a novel neuropsychiatric disorder featuring attention deficit hyperactivity disorder, severely impaired language development and normal cognition. *Genome Biol.* 17:195. 10.1186/s13059-016-1061-6 27677260 PMC5037613

[B20] Shao Z. Tumber A. Maynes J. Tavares E. Kannu P. Heon E. (2019). Unique retinal signaling defect in GNB5-related disease. *Doc. Ophthalmol.* 10.1007/s10633-019-09735-1 [Epub ahead of print]. 31720979

[B21] Simonds W. F. Zhang J. H. (2000). New dimensions in G protein signaling: G beta 5 and the RGS proteins. *Pharm. Acta Helv.* 74 333–336. 10.1016/s0031-6865(99)00043-6 10812978

[B22] Srivastava S. Love-Nichols J. A. Dies K. A. Ledbetter D. H. Martin C. L. Chung W. K. (2019). Meta-analysis and multidisciplinary consensus statement: exome sequencing is a first-tier clinical diagnostic test for individuals with neurodevelopmental disorders. *Genet. Med.* 21 2413–2421. 10.1038/s41436-019-0554-6 31182824 PMC6831729

[B23] Stankievic P. Beaudet A. L. (2007). Use of array CGH in the evaluation of dysmorphology, malformations, developmental delay, and idiopathic mental retardation. *Curr. Opin. Genet. Dev.* 17 182–192. 10.1016/j.gde.2007.04.009 17467974

[B24] Turkdogan D. Usluer S. Akalin F. Agyuz U. Aslan E. S. (2017). Familial early infantile epileptic encephalopathy and cardiac conduction disorder: a rare cause of SUDEP in Infancy. *Seizure* 50 171–172. 10.1016/j.seizure.2017.06.019 28697420

[B25] Vernon H. Cohen J. De Nittis P. Fatemi A. McClellan R. Goldstein A. (2018). Intellectual developmental disorder with cardiac arrhythmia syndrome in a child with compound heterozygous GNB5 variants. *Clin. Genet.* 93 1254–1256. 10.1111/cge.13194 29368331

[B26] Watson A. J. Aragay A. M. Slepak V. Z. Simon M. I. (1996). A novel form of the G Protein Beta Subunit Gbeta5 Is specifically expressed in the vertebrate retina. *J. Biol. Chem.* 271 28154–28160. 10.1074/jbc.271.45.28154 8910430

[B27] Zhang J. H. Pandey M. Seigneur E. M. Panicker L. M. Koo L. Schwartz O. M. (2011). Knockout of G Protein β5 impairs brain development and causes multiple neurologic abnormalities in mice. *J. Neurochem.* 119 544–554. 10.1111/j.1471-4159.2011.07457.x 21883221 PMC3192915

